# From Genesis to Old Age: Exploring the Immune System One Cell at a Time with Flow Cytometry

**DOI:** 10.3390/biomedicines12071469

**Published:** 2024-07-03

**Authors:** Anis Larbi

**Affiliations:** 1 Medical and Scientific Affairs, Beckman Coulter Life Sciences, 22 Avenue des Nations, 93420 Villepinte, France; alarbi@beckman.com; 2 Department of Medicine, Division of Geriatrics, Faculty of Medicine and Health Sciences, Université de Sherbrooke, Sherbrooke, QC J1K 2R1, Canada

**Keywords:** ontogeny, immunological memory, aging, flow cytometry, extracellular vesicles

## Abstract

The immune system is a highly complex and tightly regulated system that plays a crucial role in protecting the body against external threats, such as pathogens, and internal abnormalities, like cancer cells. It undergoes development during fetal stages and continuously learns from each encounter with pathogens, allowing it to develop immunological memory and provide a wide range of immune protection. Over time, after numerous encounters and years of functioning, the immune system can begin to show signs of erosion, which is commonly named immunosenescence. In this review, we aim to explore how the immune system responds to initial encounters with antigens and how it handles persistent stimulations throughout a person’s lifetime. Our understanding of the immune system has greatly benefited from advanced technologies like flow cytometry. In this context, we will discuss the valuable contribution of flow cytometry in enhancing our knowledge of the immune system behavior in aging, with a specific focus on T-cells. Moreover, we will expand our discussion to the flow cytometry-based assessment of extracellular vesicles, a recently discovered communication channel in biology, and their implications for immune system functioning.

## 1. Introduction

The immune system is a remarkable and intricate network of cells, tissues, and soluble mediators collaborating to shield our bodies from harmful pathogens, including bacteria, viruses, parasites, and cancer cells. Throughout our entire lifespan, from birth (and probably even before) until death, the immune system plays a pivotal role in safeguarding our health and overall well-being [1]. As we progress through life, the immune system undergoes a series of adaptations. In early life, it is still developing and adjusting to the surrounding environment. During growth, it strengthens and becomes more capable of mounting effective immune responses against a diverse array of threats. This immune system variation is due to stressful experiences individuals encounter in life. The extent of this variation, as well as specific factors shaping an individual’s immune system, stems from both heritable and non-heritable influences. However, it is the non-heritable influences, such as the presence of symbiotic and pathogenic microbes, which primarily account for most of this variation [2]. Although the immune system experiences natural wear and tear or immunosenescence with age, a gradual decline occurs in its overall function, rendering us more susceptible to infections and diseases [3].

Understanding the immune system adaptations with age will enable us to identify strategies to maintain optimal health, adding life to years [4,5]. Technological advancements have transformed the way we provide assessment and comprehension of immune system intricacies. These breakthrough technologies offer unprecedented precision and accuracy in evaluating and monitoring immune responses. To deeply map the immune system, individual cell analysis is necessary. While the bulk analysis of a population provides significant information on phenotype, functions, and roles, further understanding of population heterogeneity will likely provide detailed information on immune regulation. One such advancement involves flow cytometry, which allows us to analyze individual immune cells, providing valuable insights into their unique characteristics and functional abilities [6,7,8]. Other technologies such as single-cell RNA sequencing help delve into the genomic profile of immune cells, revealing specific gene expression patterns. These remarkable technologies empower researchers and clinicians by giving them a better immune system status understanding, abnormality identification knowledge, and personalized immune-based therapy interventions. By harnessing these advanced tools, we can unlock profound insights into the immune system, thereby paving the way for optimizing immune function strategies and combatting diseases.

In this review, we will engage in an immune system journey throughout our lives, exploring its early development and the challenges it encounters with old age. We will also examine the various factors that influence its strength and resilience, focusing on immunological history and demonstrating flow cytometry studies that have contributed to our understanding of the immune system and immunity during age. Finally, we will discuss the possible role of a recently discovered class of soluble mediators, extracellular vesicles (EVs), and how flow cytometry may help better understand their heterogeneity, as performed with immune cells.

## 2. The Immune System throughout the Lifespan

In most cases, individuals can usually respond to most pathogens following exposure; however, the exceptions of inborn immunity errors from genetic mutations and invalid responses to certain severe disease classes must be considered [9]. During life, the immune system undergoes both immunogenic and physiological changes and adapts accordingly. One example involves the reduced portion of peripheral naïve T-cells linked to thymopoiesis reduction and failed antigen-naïve T-cell production causing thymus shrinkage with age [10]. Although the peak of thymic output occurs before the fourth decade of life, this thymic or T-cell process tends to start quite early and has been a significant driver for aging research development.

Since the discovery of macrophages and phagocytosis, many scientists have proposed the role of leukocytes in the maintenance of health and longevity [11]. While the immune system undergoes crucial developmental changes from early life to adulthood, its ability to protect against infections and diseases can vary with different life stages and several contributory factors [12,13,14,15]. Various environmental factors, including exposure to pathogens, allergens, pollutants, and lifestyle choices may modulate immune fitness; moreover, vaccine development, antibiotic discovery, and hygiene improvement have increased life expectancy significantly within the past decades. Many diseases, especially infectious diseases, have been significantly reduced or even eradicated because of these practices and behaviors. Consequently, this demographic shift with advanced longevity comes with an increased prevalence of other diseases, such as cancer and neurodegenerative, cardiovascular, and other non-communicable chronic diseases, which are still often immune-related [16]. 

**Figure 1:**
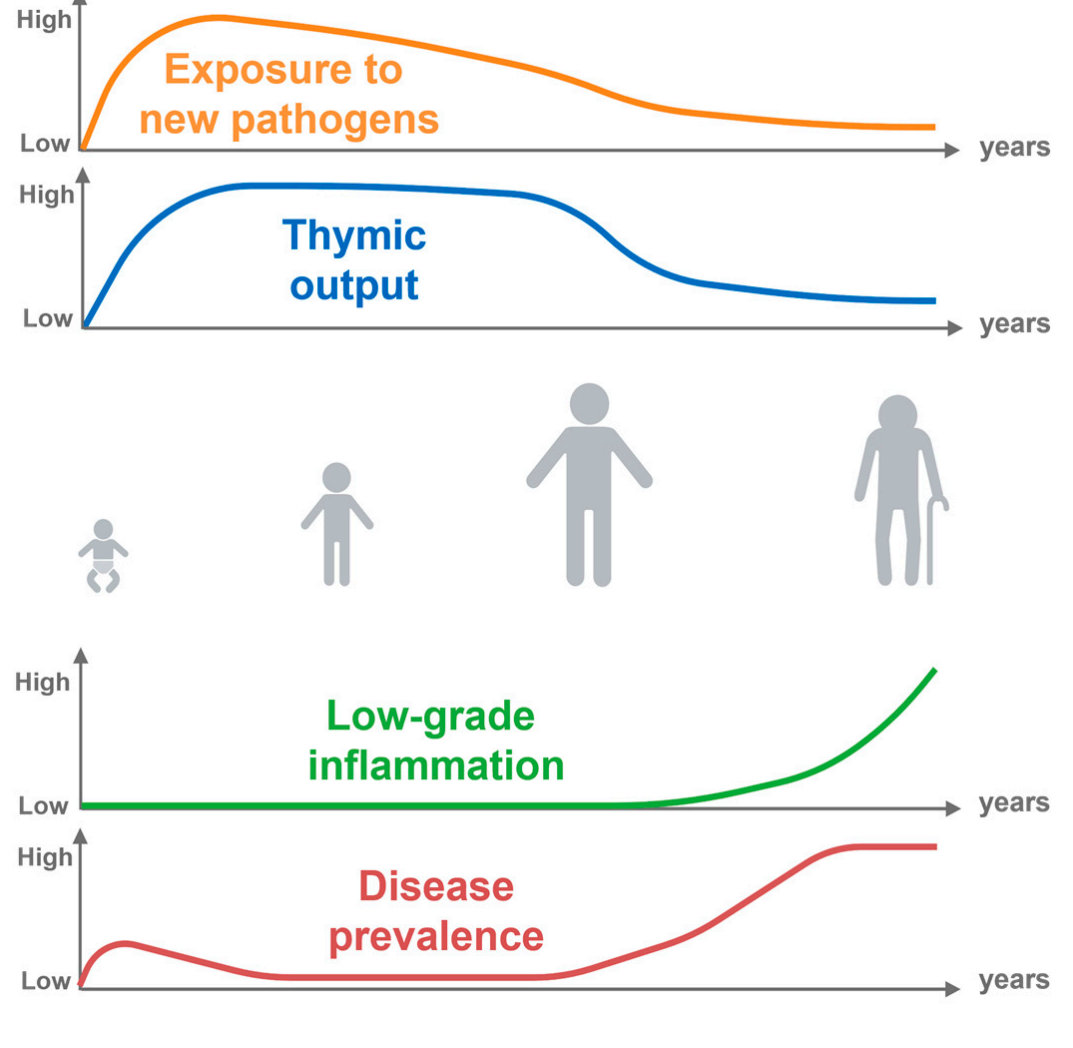
Lifelong antigen exposure. Throughout life, our exposure to new pathogens gradually increases after birth. However, the extent of this exposure is greatly influenced by environmental factors. Additionally, the production of newly generated naïve T-cells by the thymus decreases as we age. Along with these observations, the accumulation of low-grade inflammation, particularly in the later stages of life happens, which is closely linked to various diseases.

While there are many diagnostic biomarkers, none are specific for immune aging [17,18], A series of immune hallmarks which are present in most older adults can still be found. This includes a low-grade pro-inflammatory profile called inflammaging, as well as a change in the distribution of immune cell subsets, particularly the T-cell pool (Figure 1) [19,20]. Although the correlation between chronological and immune aging is not linear, evidence suggests that infants living in endemic regions with persistent cytomegalovirus (CMV) infections exhibit advanced differentiated T-cell profiles, like those observed in older adults [21]. 

Similar results apply to young adults thymectomized with an altered T-cell profile during early childhood, demonstrating further enhancement in CMV-seropositive individuals [22]. Hence, this demonstrates that studying biological markers across a lifespan is not merely time-dependent, but rather related to environmental interactions and communications, as well as responses to stress management. 

This process begins as early as pregnancy, when, in the initial weeks of conception, implantation, and early growth, the fetus requires acceptance from the maternal immune system as a separate yet tolerated system—known as tolerogenic immunity [23]. The tight balance between tolerance and protective immunity involves various cells. Organs such as the liver, bone marrow, thymus, spleen, skin, and intestine play a vital role in the development of the immune system in the fetus. Prior to the development of the hematopoietic system in the bone marrow and thymus, the initial waves of progenitors occur in the yolk sac and the extraembryonic mesenchymal tissue. Granulomacrophage progenitors and the pluripotent erythroid could be distinguished in the yolk sac at 3–4 weeks of the gestation period. At 11–12 weeks, the thymus and spleen are developed from the liver and stem cells and enable the generation of all the essential components that are required for innate and adaptive immunity. During this time, there are exchanges of crucial materials like antibodies which provide protection to the fetus against external threats. The newborn is swiftly colonized by various microbes, emphasizing the need for an effective preemptive immune response. This type of immunity, termed protective immunity, involves the gradual priming of the fetal immune system for the development of adaptive memory while still in utero [24]. Preparation of this nature allows the immune system to handle the diverse range of antigens encountered outside the protective feto-maternal environment, such as those found in breastmilk, within the gastrointestinal tract, and throughout the respiratory system. Although the newborn’s immune system must rapidly respond to a multitude of potential antigenic stimuli from the surrounding environment, it remains constantly active. Nevertheless, any compromise in its functionality is often associated with or can lead to a disease state. Manufactured compensatory mechanisms such as vaccination help a still-maturing immune system (infants) or an eroded immune system (aging or immunodeficiencies) to combat aggressions [25,26].

## 3. Decoding the Genesis of the Immune System

### 3.1. Development of the Fetal Immune System

The immune system develops to give rise to a complex network of innate and adaptive immune cells, in the periphery as well as in tissues [27,28]. The ontogeny of the immune system begins in early embryogenesis, when the first immune cells, known as hematopoietic stem cells, emerge from the mesoderm and migrate to various tissues. During pregnancy, a remarkable interplay occurs between the maternal and fetal immune systems [29]. The maternal immune system undergoes adaptations to accommodate the presence and tolerance of the developing fetus. Various mechanisms, such as immune cell regulation and the production of immunomodulatory molecules, help prevent fetal tissue rejection. Additionally, the transfer of maternal antibodies across the placenta provides passive immunity to the developing fetus, offering protection against certain pathogens. During gestation, stem cells give rise to the myeloid and lymphoid progenitor cells. The myeloid progenitors develop into innate immune cells such as macrophages, neutrophils, and dendritic cells, giving a first line of defense against pathogens to the developing embryo [30]. 

On the other hand, lymphoid progenitor cells differentiate into B-cells and T-cells, the key players of adaptive immunity [31]. While B-cells produce antibodies that target specific pathogens, T-cells orchestrate the immune response by recognizing and eliminating infected cells. Lymphocyte development involves a series of intricate processes, including gene rearrangements and thymic selection, which ensures the generation of diverse and functional immune cell populations. Fetal immune cells are produced by various hematopoietic organs in three distinct phases [1,32]. Initially, innate immune cells are generated, functioning in non-canonical roles in assisting tissue morphogenesis. Subsequently, these cells undergo further specialization to form innate immune effectors, with an ultimate phase leading to the development of adaptive immunity. Throughout this process, immune cell progression takes place in specific tissues, such as the concentration of memory T-cells occurring within the gut. However, it is important to note that the simultaneous activation of innate immunity alongside immune cell progression can have detrimental effects on fetal development. As the pregnancy progresses into the second trimester, the presence of regulatory T-cells becomes crucial in maintaining a balanced and developed adaptive immune system. Overall, the development of both innate and adaptive immune cells is crucial for establishing a robust and effective immune response throughout an individual’s lifespan [33]. 

### 3.2. Myeloid Cell Ontogeny

During fetal development, the ontogeny of myeloid cells is a fascinating process that involves the differentiation and maturation of various cell types as well as the emergence of hematopoietic stem cells from the mesoderm. Although these stem cells have the potential to generate all the blood cell lineages, they migrate to the fetal liver, where they undergo expansion and differentiation [34]. During progression, the fetal liver serves as a crucial site for the emergence and differentiation of myeloid progenitor cells derived from hematopoietic stem cells. These myeloid progenitor cells give rise to diverse subsets of myeloid cells, including macrophages, neutrophils, dendritic cells, and erythrocytes [35]. Macrophages, with their multifunctional roles in phagocytosis, antigen presentation, and tissue homeostasis, originate from the monocyte precursors that infiltrate tissues and subsequently differentiate into tissue-resident macrophages [36]. Neutrophils, renowned for their innate immune defense against bacterial infections, also undergo fetal stage development; however, neutrophil precursors are initially generated within the fetal liver and later migrate into the bloodstream [37]. Similarly, dendritic cells are also derived from monocyte precursors and play a pivotal role in immune surveillance and antigen presentation. These cells also display distinct subsets in various tissues, such as the skin, mucosa, and lymphoid organs [38]. Additionally, erythrocytes belong to the myeloid lineage, provide oxygen transport, and undergo multiple maturation stages within the liver before entering circulation [39]. It is evident that the fetal liver plays a crucial role in the ontogeny of myeloid cells, contributing to the establishment of a functional innate immune system during early development [40]. Moreover, it is crucial for the establishment of the innate immune system and the overall vitality of fetal health and development.

### 3.3. Lymphoid Cell Ontogeny

**Figure 2:**
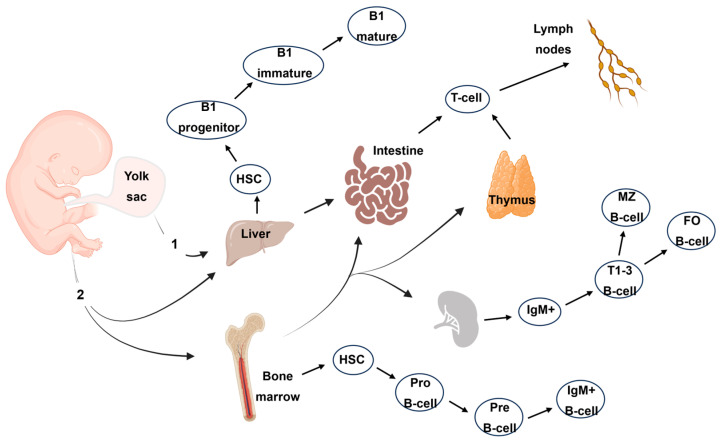
Lymphocyte cell production in the human embryo. The main routes include primitive hematopoiesis (1) in the first weeks of gestation within the human yolk sac. After five weeks of gestation, the hematopoietic progenitors initiate definitive hematopoiesis (2). Only T- and B-cell production is depicted here. HSC: hematopoietic stem cells; MZ B-cell: marginal zone B-cell; FOB-cell: follicular B-cell.

During fetal development, the rise of lymphoid cells is orchestrated by a complex and meticulously regulated process [41]. Without functional bone marrow and thymus, the first transient waves of hemopoiesis occurred in the mesoderm of the yolk sac (Figure 2). These stem cells migrate to the fetal liver and to the thymus, where they differentiate and undergo further maturation. The lymphoid progenitors migrate from the liver to the thymus at eight weeks of gestation [42,43]. Within the thymus, T-cell maturation entails a series of intricate steps, encompassing gene rearrangements and selection processes. The progenitor cells within the thymus differentiate into immature T-cells and undergo positive and negative selection mechanisms, forming a diverse and functional T-cell generation repertoire [44]. At 10–11 weeks, the thymus is functional. T-cells are present before birth with regulatory T-cells that suppress cytokine secretion from other fetal T-cells [45,46]. 

Bone marrow formation occurs around eight weeks of gestation and contains hematopoietic progenitors and stem cells [47]. The bone marrow is the source of B-cells which are very prevalent in the spleen at mid-gestation [48]. While originating from B-1 progenitors at the early stage of pregnancy, the B-2 progenitors are predominant at the end of fetal life [49]. The maturation of B-cells involves the rearrangement of immunoglobulin genes and the selection of proficient B-cells that possess the ability to recognize a wide range of antigens. Within the fetus’s peripheral circulation, B-cells appear as early as 12 weeks. While the ontogeny of lymphoid cells entails a highly regulated and intricately coordinated series of events, it is paramount for the establishment of a functional adaptive immune system, ensuring the generation of diverse and competent T- and B-cell populations during fetal development. Overall, these cells act as sentinels, perpetually engaged in immune surveillance and defense against pathogens throughout an individual’s lifespan.

### 3.4. Soluble Factors of Immunity during Fetal Development

During fetal development, the production and function of antibodies, cytokines, and the complement system play pivotal roles in the immune defense of the developing fetus. Antibodies, also known as immunoglobulins, are specialized proteins generated by B-cells. In the early stages of fetal development, the fetus acquires a limited supply of antibodies from the mother through passive transfer across the placenta. These maternal antibodies provide transient protection against specific pathogens until the fetus can independently produce its own antibodies. As fetal B-cells mature, they begin synthesizing their unique antibodies. At 10 weeks of gestation, the spleen is the early IgG and IgM supplier; however, IgG levels increase significantly until birth while IgE is detected in the fetal liver and spleen at 11 and 21 weeks of gestation, respectively [50,51]. Cytokines, small proteins secreted by immune cells, regulate various immune processes and contribute to cell growth, differentiation, and the coordination of immune responses during fetal development. While little is known about fetal cytokine production, there is clear evidence regarding the role of regulatory maternal cytokines for pregnancy outcomes [52,53]. 

The complement system, a group of proteins that amplify the immune response, undergoes development and aids in pathogen destruction and immune complex clearance [54]. Although the complete functionality of the complement system may not be reached until after birth [55], its components actively engage in immune processes during fetal development. Collectively, antibodies and the complement system collaborate to fortify the defense mechanisms of the developing fetus, affording protection against potential infections.

## 4. The Temporal Dimension for the Immune System

At birth, the immune system is still in its early stages of development and is considered relatively immature [56]. However, it rapidly adapts to the new environment and begins to function independently. Initially, the neonatal immune system relies heavily on innate immune responses, such as phagocytosis and complement activation, to combat infections. Over time, adaptive immune responses, including the development of memory cells and specific antibody production, gradually develop and strengthen. Since early life poses unique challenges to the developing immune system, vaccination strategies must be adapted for this fragile population [57]. While neonates are particularly susceptible to infections due to their immature immune responses and the limited transfer of maternal antibodies, they also face exposure to a wide range of pathogens, both in the hospital and within community settings. Common infections in early life include respiratory, gastrointestinal, and skin infections [58]. These challenges serve as important stimuli for immune maturation and the establishment of immune memory.

In response to pathogens, the generation of T-cell memory serves as a pivotal aspect of the immune response, greatly impacting long-term protective immunity [59]. Following a pathogen encounter, specific T-cells are activated and undergo clonal expansion, mounting a targeted defense against the infection. Within this process, a distinct subset of T-cells differentiates into memory T-cells, characterized by unique attributes allowing them to confer durable immunity. These memory T-cells exhibit heightened responsiveness and rapid activation upon re-exposure to the same pathogen. Moreover, they possess the remarkable capacity to swiftly proliferate and differentiate into effector T-cells, which exhibit enhanced pathogen elimination compared to the primary immune response. Although T-cell classification is ever evolving, they are usually classified as antigen-naïve (T_NAIVE_), central memory (T_CM_), effector memory (T_EM_), and terminal effector (T_TE_) cells [60]. The generation of T-cell memory relies on intricate interactions between T-cells and antigen-presenting cells, along with the presence of specific cytokines and costimulatory signals. This process ensures the retention of immunological “memory” from prior pathogen encounters, facilitating a prompt and robust immune response upon subsequent exposures, which is key to sustaining a healthy lifespan [61].

**Figure 3:**
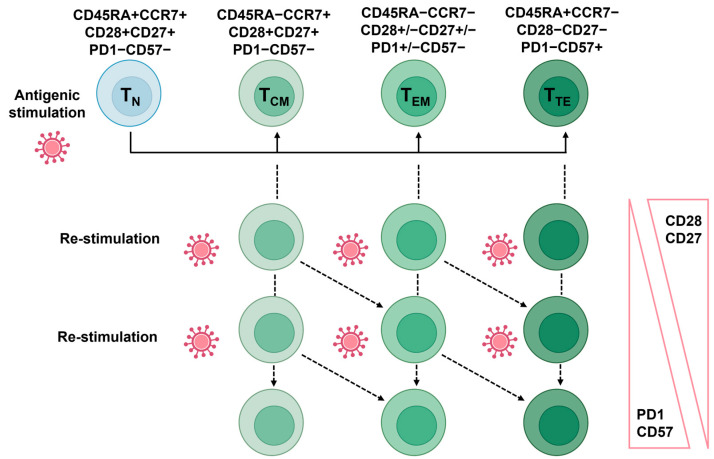
Representation of the main T-cell subsets and their respective markers. The antigenic stimulation will initiate the production of memory cells (central memory, effector memory, or terminal effector). Subsequent stimulation by the same antigen will lead to the further differentiation of the memory subset to the next stage. Alternatively, the memory cells will retain their subset classification but gain or lose senescence/exhaustion or co-stimulatory molecules, respectively. The markers used to classify the four main subsets depicted here may vary depending on the antigenic stimulation.

While memory is one of the greatest features of immunity, it can also lead to memory inflation. For example, persistent CMV infections pose a unique challenge to the immune system because they can cause complete elimination and establish a state of chronic infection allowing certain immune cells, particularly CD8+ T-cells, to become persistently stimulated by viral antigens [62]. These CMV-specific CD8+ T-cells undergo a series of phenotypic and functional changes, collectively referred to as the aforementioned “memory inflation”. Over time, the frequency of CMV-specific T-cells increases in response to continuous antigen exposure [63]. These cells also acquire a distinct phenotype, characterized by the expression of specific surface markers and the production of effector molecules (Figure 3). Certain changes in the expression of T-cell CD28 and CD27, a co-stimulatory molecule critical for T-cell activation, tend to accompany T-cell differentiation. With advancing age, there is a lower expression of both molecules, leading to the accumulation of CD28-CD27- T-cells, also known as “senescent” or “late-stage differentiated” T-cells [20,64]. These cells exhibit reduced proliferative capacity, shortened telomeres, and altered signaling pathways. 

The CD57 marker is both a T-cell aging differentiation and replicative senescence marker. With age, CD57 expression increases in memory T-cell subsets and is commonly found on CD8+ T-cells [65]. Additionally, the expression of certain inhibitory receptors, such as PD-1, more related to immune exhaustion, is upregulated in T-cells in older individuals [66]. These markers reflect the phenotypic and functional changes that occur during T-cell differentiation in aging, ultimately impacting the immune response and contributing to immune senescence. Understanding the aging markers expressed during T-cell differentiation is crucial for elucidating age-related immune dysregulation and developing strategies to enhance immune function in older individuals. Similar observations were made for other lymphoid cells such as B-cells and NK cells [67,68]. Several studies also demonstrated the erosion of innate immunity with age including neutrophils, macrophages, monocytes, and dendritic cells [69,70,71,72].

Inflammaging, a term used to describe the chronic, low-grade inflammation that occurs with aging, involves a persistent activation of the immune system and an increase in pro-inflammatory markers within the body [73]. During aging, a combination of factors including cellular senescence, immunosenescence, oxidative stress, and immune system dysregulation contributes to the development of inflammaging. This chronic inflammation has been associated with a variety of age-related diseases, such as cardiovascular disease, neurodegenerative disorders, metabolic disorders, and cancer [74]. Furthermore, inflammaging is linked to accelerated aging and a decline in overall physical and cognitive function as well as to senescence-induced secretory phenotype (SASP) [75,76]. SASP defines the secretory profile of senescent cells, which includes cytokines, chemokines, proteases, and growth factors. While aging is associated with an increased number of senescent cells, including immune cells, the circulating milieu is likely influenced and may support the development of diseases [77]. Significant work is still warranted to understand the mechanisms and consequences of inflammaging and SASP, thereby enabling the development of effective strategies to mitigate effects and promote healthier aging. 

## 5. The Contribution of Flow Cytometry to Understanding Immunity during Lifespan

### 5.1. Principles of Flow Cytometry

**Figure 4:**
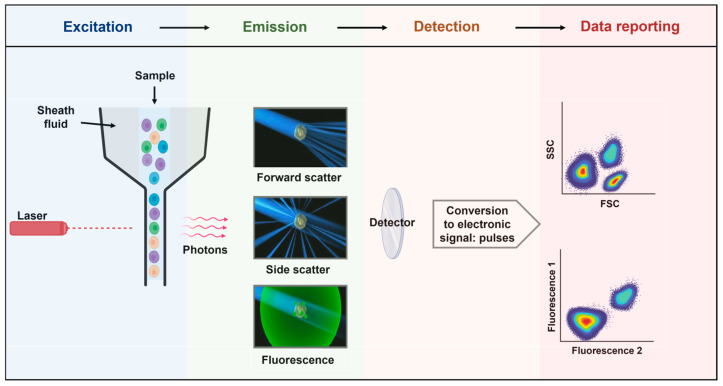
Principles of flow cytometry. Flow cytometry is a technology used to analyze the physical and chemical characteristics of particles in a fluid (sheath fluid) as they pass through at least one light source (lasers). The light detected (photons) is converted into pulses that are subsequently displayed in plots, histograms, or other visualizations. Although the fluidics system may differ (pressure, syringe, peristaltic pump, and vacuum), the optics (excitation and emission collection) are composed of lasers, the flow cell, filters, and detectors.

Flow cytometry is a robust analytical technique extensively employed in biomedical research and clinical diagnostics. It operates with light scattering and fluorescence principles, enabling the examination of individual cells or particles within a liquid medium [78]. The fundamental principles of flow cytometry encompass the precise detection and measurement of scattered light and emitted fluorescence, the capacity to simultaneously analyze multiple parameters, and the sorting of cells or particles based on their unique characteristics [79]. Flow cytometry enables the measurement of particles and cells by both size and fluorescence [80]. With the advent of a light source (lasers), the biological sample is interrogated cell by cell through the flow. The light emitted and scattered is then collected, converted into an electronic signal, and displayed for analysis (Figure 4). 

Leonard Herzenberg, a true pioneer in the field of flow cytometry, made groundbreaking contributions that revolutionized biomedical research and diagnostics [81]. By adding antibodies targeting known receptors and a fluorescent secondary antibody as a reporter, he could visualize different immune cell populations in a simple way. This innovative work laid the foundation for the subsequent advancements in flow cytometry, including the development of monoclonal antibodies and the integration of multiple parameters analysis. To date, dozens of fluorochromes exist and can be combined for high-dimensional flow cytometry analysis, maximizing the information generated from the same sample. By leveraging these principles, researchers and clinicians can interrogate essential cellular attributes such as cell size, granularity, and the expression of proteins, signaling molecules, and even mRNA [82]. 

### 5.2. The Contribution of Flow Cytometry in Immunology

Flow cytometry has emerged as an invaluable tool in unraveling the complexities of immune responses [83,84]. This technique allows for the simultaneous analysis of multiple parameters, encompassing cell surface markers, intracellular cytokines, and functional assays, enabling a comprehensive characterization of immune cell populations [85,86,87,88]. Through the precise identification and analysis of distinct immune cell subsets, such as T-cells, B-cells, and natural killer cells, flow cytometry has provided profound insights into their diversity and functional capabilities [89,90]. It has greatly enhanced immune cell development and differentiation understanding, as well as the intricate responses to various stimuli such as pathogens, vaccines, and diseases [91,92]. 

The diagnosis and management of HIV-positive patients has been one of the first and obvious contributions utilizing flow cytometry, allowing clinicians to accurately assess and monitor the immune status of HIV-infected individuals [93]. This technique allows for the quantification and characterization of various immune cell populations, such as CD4+ T-cells and CD8+ T-cells, which are crucial in evaluating immune function [94]. Flow cytometry enables the measurement of absolute CD4+ T-cell counts and the determination of the CD4/CD8 ratio, which are essential indicators of disease progression and the effectiveness of antiretroviral therapy (ART) [95]. Additionally, flow cytometry helps identify activation markers and assess immune activation levels, providing valuable insights into the immune response and potential complications associated with HIV infection. With its ability to provide detailed immunophenotyping data, flow cytometry plays a significant role in guiding treatment decisions, monitoring disease progression, and predicting clinical outcomes, especially for leukemia/lymphoma [96,97,98,99]. Since the field of flow cytometry is constantly evolving, fewer drops of blood can now be used to discriminate many more immune cell populations [84]. With the advent of flow cytometry, not only has the classical definition of T-cell subsets (T_N_, T_C_, T_EM_, and T_TE_) significantly developed, but the family of CD3+ T-cells has also been further deciphered [100,101,102]:

- Naïve T-cells that recently emigrated from the thymus: T_RTE_ cells express CD31.
- Antigen-naïve T-cells: T_N_ with no antigenic experience but homeostatic replicative history.
- Virtual-memory T-cells: T_VM_ have a memory phenotype prior to antigenic contact.
- T-cells with stem cell-like properties: T_SCM_ cells possess the highest proliferative capacity of memory cells; they express CD95, CXCR3, CD45RA, CCR7, and CD27.
- Central memory T-cells: T_CM_ express the lymph node homing molecules and have limited effector functions.
- Effector Memory T-cells: T_EM_ preferentially traffic to peripheral tissues and mediate rapid effector functions.
- Transitional Memory T-cells: T_TM_, defined as CD45RA-CCR7-CD28+, have an intermediate differentiation status between CM and EM.
- Terminal Effector T-cells: T_TE_ are memory cells re-expressing CD45RA.
- T helper 1: Th1 producing IFN-γ.
- T helper 2: Th2 producing IL-4, IL-5, IL-13, and IL-9.
- T helper 17: Th17 producing IL-17 and IL-22.
- Regulatory T-cells: Treg expressing FoxP3 which are suppressor functions.
- Follicular T-cells: Tfh producing IL-21 and expressing CXCR5.

To date, flow cytometry in used in various clinical cases as an indispensable tool for immune monitoring across a range of clinical scenarios, including cancer immunotherapy, autoimmune diseases, and other immunological disorders. In the realm of cancer immunotherapy, flow cytometry plays a crucial role in characterizing and quantifying immune cell subsets, such as tumor-infiltrating lymphocytes (TILs) and immune checkpoint molecules [103,104]. This enables researchers and clinicians to gain valuable insights into the tumor microenvironment and assess the effectiveness of immunotherapeutic interventions. By evaluating immune cell activation, proliferation, and cytokine production, flow cytometry aids in monitoring treatment response and identifying potential biomarkers for personalized therapies. 

In the context of autoimmune diseases, flow cytometry facilitates the analysis of immune cell profiles and functional assays, shedding light on immune system dysregulation and identifying specific cell populations associated with disease pathogenesis [105]. By measuring surface markers and intracellular cytokines, flow cytometry helps to characterize aberrant immune cell subsets, such as autoreactive T-cells and regulatory T-cells, supporting the development of targeted therapies and monitoring treatment efficacy. It also assists with monitoring immune responses in transplantation, infectious diseases, allergic reactions, and immunodeficiency disorders, as well as helping with immune cell identification and quantification, cytokine production assessment, and immune marker activation measurements [106]. While these insights provide valuable information for diagnosis, prognosis, and therapeutic decision making, the widespread application of flow cytometry for immune monitoring in diverse clinical contexts has played a pivotal role in advancing the understanding of immune dysregulation and facilitating the development of personalized and targeted therapeutic strategies [107,108,109,110].

### 5.3. The Analysis of the Human Immune System in Aging

#### 5.3.1. Immunosenescence

Immunosenescence refers to the gradual deterioration of the immune system, particularly in older individuals, over time. All the immune cells display age-related adaptations [111,112,113,114]. One key aspect of immunosenescence is the impact that it has on T-cells, which tends to be the focus of this review. With age, T-cells undergo various changes that contribute to their functional decline. These changes include a decrease in the diversity of the T-cell receptor repertoire, a shift in T-cell subsets toward more differentiated and memory cells, and a decline in the production of naïve T-cells from the thymus [3]. Additionally, T-cells in older individuals exhibit reduced proliferative capacity, impaired cytokine production, and altered signaling pathways. These age-related alterations in T-cells compromise their ability to mount effective immune responses, leading to increased susceptibility to infections, reduced vaccine responses, and a higher risk of developing chronic inflammatory diseases [115].

Understanding the mechanisms underlying T-cell immunosenescence is crucial for developing interventions to enhance immune function and promote healthy aging. This is particularly relevant during the course of life, as immune adaptations are likely to (i) compromise the immune system’s ability to mount effective immune responses against pathogens, leading to an increased susceptibility to infections, including viral, bacterial, and fungal infections [116]. Older individuals may experience more severe and prolonged infections, contributing to higher morbidity and mortality rates while (ii) impacting the effectiveness of vaccinations, resulting in reduced vaccine responses in older individuals [117]. This has implications for immunization programs and strategies to prevent infectious diseases because older adults may have diminished abilities to generate protective immune responses against vaccines yet (iii) impair the immune system’s ability to detect and eliminate cancer cells [118]. This may contribute to an increased risk of developing certain types of cancers and a reduced response to cancer therapies (iv) contributing to an increased risk of autoimmune disorders [119]. The dysregulation of the immune system in older individuals may result in the loss of self-tolerance, leading to the development of autoimmune conditions such as rheumatoid arthritis, systemic lupus erythematosus, and type 1 diabetes (v), associating the conditions with a chronic low-grade inflammatory state, often referred to as inflammaging [120]. This chronic inflammation can contribute to the onset and progression of age-related diseases.

#### 5.3.2. Markers of Immunosenescence

**Figure 5:**
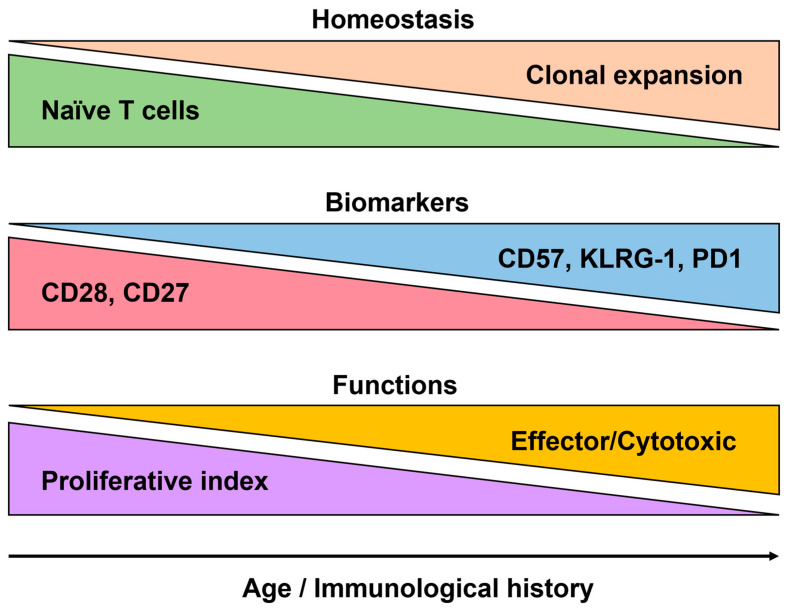
The main features of T-cell senescence. The major changes observed at the homeostatic level show the consensus markers as well as the functional changes with aging and/or associated with immunological history.

Several markers and functional characteristics are associated with immunosenescence, particularly for T-cells. One marker commonly associated with immunosenescence is CD28, which is a co-stimulatory molecule that plays a crucial role in T-cell activation. During aging, the expression of CD28 on T-cells declines, leading to the accumulation of CD28- T-cells (Figure 5). These CD28- T-cells are often characterized by shorter telomeres, indicating a higher replicative history, and CD27 tends to follow the same path of expression/downregulation as seen in T-cells [121]. The loss of these markers on T-cells during aging is indicative of functional impairments and a shift toward more differentiated and senescent T-cell subsets. Replicative senescence is highly linked to the expression of CD57 and other senescence markers such as KLRG-1 [122]. Recent studies on CD8+ T-cells show the expression of NK-like markers at the last stages of T-cell differentiation [123]. Functionally, immunosenescent T-cells exhibit decreased proliferative capacity and impaired cytokine production. They often have reduced antigen-specific responses and a diminished ability to clear infections while showing altered signaling pathways, including defects in the activation of transcription factors such as NF-κB and downstream effector molecules. Immunosenescent T-cells also display an altered balance between pro-inflammatory and anti-inflammatory cytokines [124,125]. There is a shift toward a pro-inflammatory profile, with the increased production of inflammatory cytokines such as interleukin-6 (IL-6) and tumor necrosis factor-alpha (TNF-α), and the reduced production of anti-inflammatory cytokines like interleukin-10 (IL-10).

Not only did the early studies using flow cytometry show a shift in the phenotype and functions of CD4+ T-cells with aging but this was also indicative with CD8+ T-cells [126,127]. Reduced T-cell receptor (TCR) diversity and impaired T-cell proliferation were also observed in older individuals along with an accumulation of CD8+ T-cells with shortened telomeres, a marker of cellular aging [128]. Since then, a series of reports have shown that inflation of the memory pool of T-cells could be linked to some persistent infections such as cytomegalovirus (CMV); consequently, immunological aging is highly influenced by infection history. 

This was nicely demonstrated by Miles et al., showing that CMV+ infants could display a persistent immune profile similar to older adults, although CMV is not the only model to demonstrate the impact of infection history on the immune profile [129]. Studies by Appay et al. revealed that HIV+ individuals also induce a premature aging of the immune system, additionally to the impact on the CD4+ T-cell counts [130]. More recently, Kared et al. utilized advanced techniques such as high-dimensional flow cytometry and single-cell RNA sequencing to uncover the underlying factors contributing to the heterogeneity of T_SCM_ [131]. They found that this heterogeneity is influenced by the differential activation of Wnt signaling pathways [132]. Interestingly, the authors observed that the aging process in humans is associated with a decline in the Wnt/β-catenin signature in CD4 T_SCM_. Additionally, they noted a systemic increase in the levels of Dickkopf-related protein 1, a natural inhibitor of the Wnt/β-catenin pathway. Furthermore, the study revealed that T_RTE_ are the preferred precursors of CD4 T_SCM_, suggesting that metabolically targeting the Wnt/β-catenin pathway could potentially reverse TSCM defects and possibly restore and maintain immune homeostasis. 

Quinn et al. showed that the proliferative capacity of T_VM_ cells, which are highly proliferative in young individuals, significantly decreases in aged mice and humans [133]. Interestingly, conventional naïve T-cells retain their proliferative capacity in both aged mice and humans. To investigate the underlying mechanisms, adoptive transfer experiments in mice revealed that naïve CD8 T-cells could acquire a proliferative defect when exposed to the aged environment. However, it was observed that the age-related proliferative dysfunction could not be rescued by providing a young environment [134]. The molecular analyses of the aged TVM cells unveiled a profile consistent with senescence, marking a significant observation of senescence in an antigenically naïve T-cell population. 

Overall, the markers and functionality of immunosenescence in T-cells reflect a decline in their ability to mount effective immune responses. This mechanism starts with the recently emigrant T-cells from the thymus, virtual memory T-cells with no antigenic history, T_SCM_, and concludes with the last stages of differentiated T-cells. By quantifying specific immune cell populations and examining functional parameters using techniques such as flow cytometry, scientists have provided insights into the impact of aging and immunological history on immune fitness.

#### 5.3.3. Exploring Aging through Extracellular Vesicles (EVs)

As mentioned above, the environment in which immune cells evolve strongly influences their function. Quinn et al. showed that naïve CD8 T-cells could acquire a proliferative defect when exposed to the aged environment. Soluble factors are then a suitable target for restoring immune function in old age. While inflammaging and SASP have shown their value in our understanding of immune aging, a recently discovered communication channel, extracellular vesicles (EVs), have gained significant attention [135,136]. This is primarily due to their potential role in intercellular communication and their involvement in diverse physiological and pathological processes [137,138]. 

**Figure 6:**
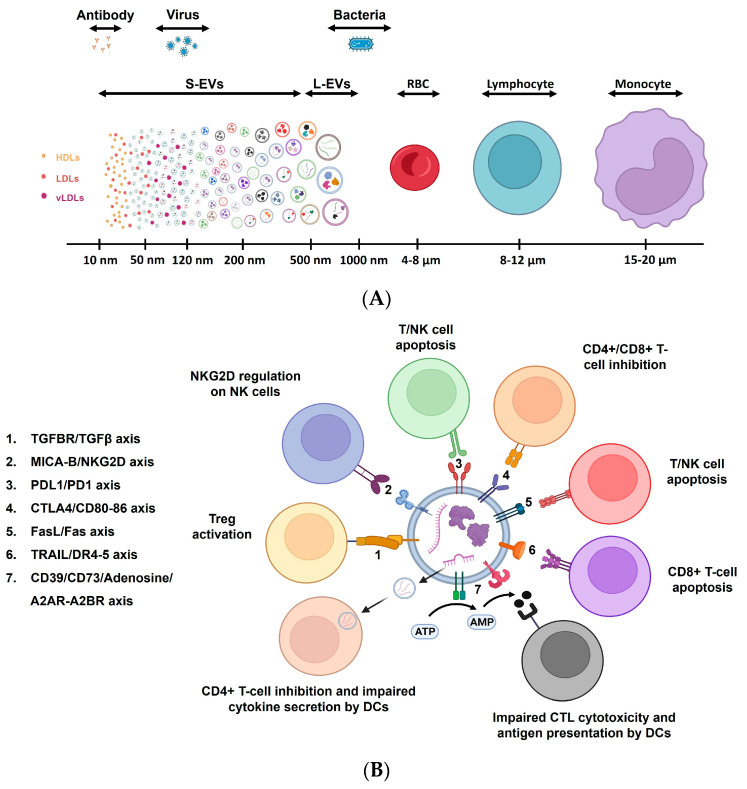
Extracellular vesicles in immune regulation. (**A**) The size distribution and heterogeneity of EVs are depicted. Immune cells and other entities such as antibodies, bacteria, and red blood cells are shown to compare size. (**B**) The regulatory molecules expressed on extracellular vesicles modulate the activity of immune cells. Cytokines and other molecules such as ATP regulate the EV-immune cell interactions. Modified from [149]. DC: dendritic cells; NK: natural killer cells; CTL: cytotoxic T lymphocytes.

EVs are small membrane-bound vesicles (30–500 nm) that carry a cargo of proteins, nucleic acids, lipids, and other molecules when released by cells into the extracellular space (Figure 6A) [139]. In aging, the production, composition, and function of EVs are influenced by the increasing number of senescent cells [140]. For example, EVs obtained from hindlimbs were more widespread in a sarcopenic mice model. These EVs were found to be enriched in miR-335-5p, miR-320a, miR-483-5p, and miR-21-5p, both in mice and humans, denoting that EVs derived from aged cells may exhibit changes in their cargo, including the altered levels of specific miRNAs, proteins, and lipids [141,142]. Age-related changes of this nature can influence the functional properties of EVs, such as their ability to modulate immune responses, cellular senescence, and tissue repair mechanisms [143,144]. 

EVs derived from aged cells have been implicated in age-related pathologies, including neurodegenerative diseases, cardiovascular disorders, and age-related macular degeneration [145]. For example, data indicate that bone marrow-derived mesenchymal stem cell (BM-MSC)-derived EVs in young BM-MSC individuals tend to be more effective in promoting bone regeneration compared to EVs from aged BM-MSCs [146]. EVs derived from aged retinal pigment epithelial cells (RPE) were shown to contain higher levels of specific miRNAs that induced senescence in recipient RPE cells, suggesting that EV-mediated miRNA transfer contributes to the senescence-associated changes observed in age-related macular degeneration [147]. 

To test the feasibility of EVs to restore cellular functions in old age, EVs derived from mesenchymal stem cells (MSCs), in a model of Hutchinson–Gilford progeria syndrome, were used. The researchers found that treatment with MSC-derived EVs could reverse some signs of aging [148]. These studies highlight the involvement of EVs in aging and age-related diseases, shedding light on their role in cellular communication and disease pathogenesis. Understanding the role of EVs in aging and age-related diseases holds promise for developing novel diagnostic tools and therapeutic strategies to promote healthy aging and mitigate age-associated pathologies.

#### 5.3.4. Immunity, Aging, and Extracellular Vesicles

Studies focusing on the potential role of EVs in the modulation of the immune system are emerging, including their use in vaccine development, their role in immune regulation, and response to infectious diseases in the context of immunotherapy, based on the fact that EVs have been shown to interact with various immune cell populations [149,150,151,152]. The components of the innate and adaptive immune system are influenced by EVs (Figure 6B). Through molecules expressed on their surfaces, EVs bind to corresponding receptors expressed by the immune cells and directly affect intracellular signaling and further influence cell fate and behavior. Hence, effector cell activation, regulatory functions, apoptosis, and antigenic presentation capacity are influenced by the interaction of EVs with immune cells. 

Recent studies have revealed that EVs can contribute to the hypoxia-induced immunosuppressive effects, affecting tumors and immune cells. Hypoxic zones within glioblastoma (GBM) tumors are known to be associated with poor prognosis and immunosuppression. Hypoxia was shown to enhance the release of EVs from cells and upregulated the expression of miR-25/93 [153]. Macrophages engulfed the hypoxic EVs that contained miR-25/93. Consequently, the activation of the cGAS-STING pathway, which is crucial for type I interferon (IFN) expression and secretion by macrophages, was impaired. Macrophages treated with these EVs downregulated M1-associated genes. This downregulation resulted in a reduced number of IFN-γ+ T-cells attracted. This example of the immunoregulatory role of EVs highlights their involvement in the series of immune response events.

Because EVs may regulate immune responses, we could hypothesize that changes in their numbers and characteristics in aging may contribute to immune erosion and could be used as a therapeutic target to boost immunity in later life [154]. A series of reports tend to validate the hypothesis of a potential role of EVs in immune dysregulation during aging [139,149,155]. The effect of age on the inflammatory profile of EVs was demonstrated and could be linked to the senescence-associated secretory phenotype (SASP) and inflammaging [156,157]. With advanced age, there is an increased load of pro-inflammatory miRNAs in EVs derived from mesenchymal stem cells. EVs derived from senescent cells cause a loss of homeostasis and can lead to dysregulated inflammation [158]. Furthermore, EVs extracted from young mice serum were able to attenuate inflammaging in old mice, partially contributing to the rejuvenation of thymic aging, as well as highlighting how EVs could contribute to age-associated mechanisms contributing to disease onset and progression [159]. Immune cells are involved in the process of senescent cell removal, and it was shown that perforin −/− mice were not able to perform this task properly, suggesting that alteration in immune profiles with aging may affect the regulation of pro-inflammatory senescent cell-derived EVs [160]. This raises the question of whether or not EVs could be directly involved in immunosenescence and reduced immune response in aging. Based on the immunoregulatory role of EVs, it is highly possible that alterations in the number and characteristics of EVs (surface markers and cargo) will affect homeostasis and immune fitness [149]. Further studies will be needed to validate this hypothesis.

One limitation for immunologists to decipher the complexity of the interactions between EVs and the immune system pertains to the ability to analyze EVs in the same way they currently analyze immune cells. First, the analysis of EVs requires a series of tools. For example, the isolation of EVs may be performed by ultracentrifugation or size-exclusion chromatography and then checked for purity and concentration [161]. Once this is performed, the characterization can be performed. Depending on the research conducted, various alternatives exist, such as electron microscopy, genomic analysis, and lipidomics or proteomics. 

While Western blotting and ELISA have been used historically, they do not provide insight into the heterogeneity of the sample. Analyzing EVs individually would be an ideal way to move forward within the biomarker field utilizing flow cytometry, which analyzes events one by one [162]. This is conditioned by the ability of the cytometer to detect small particles, although it was originally designed to analyze much larger events such as cells or bacteria [163]. By increasing the sensitivity of cytometers, Witwer et al. showed that it was possible to detect EVs [164]. Flow cytometry was used to analyze EV size distribution, surface marker expression, and the presence of specific EV-associated proteins, allowing for a comprehensive analysis of EV populations using different isolation techniques. 

Protocols have been developed to overcome some of the technical issues such as swarming and impurities [165]. By using a combination of size and fluorescence-based markers, it is possible to address the issue of protein aggregates. Flow cytometry was used to analyze EV populations and exclude non-vesicular particles, enabling a more accurate characterization of EVs in plasma samples. For instance, Renner et al. demonstrated the effectiveness of flow cytometry to differentiate Moloney murine leukemia virus from EVs [166]. With the advent of more sensitive and modified optical benches, it is also possible to resolve particles as small as 80 nm [167]. Recently, an extensive review of these aspects has become available to the community of scientists to improve their analysis of EVs by flow cytometry [161,162]. Since the size of EVs can be lower than 80 nm, being able to detect and characterize the smaller EVs (<80 nm) would provide higher accuracy for determining the role of EVs in immune regulation. Overall, this suggests a promising approach to studying soluble factors like EVs and the manner in which they influence immune responses. Moreover, these applications could bring significant insights into cancer dissemination mechanisms and immune response boosting [168,169,170,171]. 

## 6. Conclusions

The immune system is a complex network of interactions which involves multiple cell types. While this review focused on T-cells, many cell types display age-related adaptations, including B-cells, NK cells, neutrophils, and macrophages. Technological progression with flow cytometry has enabled deciphering the complexities of the immune system from in utero development to old age. This understanding helps to address key issues such as hypo-responsiveness through the lifespan. Understanding these defects has resulted in better strategies for vaccination, especially for influenza. The recent discovery of EVs and their potential role in the regulation of the immune system is opening new avenues for scientists. With the advent of nano flow cytometry, a portion of the unknown could be revealed and help identify alternative strategies to support immune fitness in old age. The most significant limitation of flow cytometry is the ability to detect the smallest EVs (<80 nm). Nanoparticle tracking analysis (NTA) is one of the most popular techniques to control for the number and size of EVs; however, the settings need to be adapted to the type of sample and size of EVs, which is not always predictable [172]. Another limitation of NTA is its limitation to characterize the phenotype of EVs, which can be resolved by flow cytometry. Dynamic light scattering (DLS) measures the intensity of scattered light as a function of time and enables us to resolve size distribution but not the profile of EVs. Finally, electron microscopy is probably the best tool to define the morphology and best sizing method for EVs; however, the very low throughput is a limitation, and it does not enable us to characterize the phenotype of EVs. Studies still need to address whether the use of flow cytometry alone could replace other orthogonal methods for the counting and characterization of EVs. As EVs are slowly entering into clinical trials, designing EVs to target specific immune cells and boost immunity could be envisioned [173]. This would apply not only to support immunity against infectious agents but also against the rising cases of cancers.
